# Inter-Generational Differences in Perinatal Health Behaviours: A Secondary Analysis of the Born in Bradford Cohort, Disentangling Ethnicity and Migration

**DOI:** 10.1007/s10995-023-03637-0

**Published:** 2023-05-10

**Authors:** Katie Marvin-Dowle, Hora Soltani

**Affiliations:** Faculty of Health and Wellbeing, College of Health, Wellbeing and Life Sciences, Sheffield Hallam University, Collegiate Crescent, Sheffield, S10 2BP United Kingdom

**Keywords:** Pregnancy, Perinatal, Migration, Health behaviours, Ethnicity, Mental Health

## Abstract

**Objectives:**

There exists a body of research regarding ethnic differences in perinatal health whereas this is not the case concerning the role of migration status and acculturation in attenuating these differences. This study aims to investigate determinants of health during pregnancy up to one-year postpartum by migration status.

**Methods:**

The study utilises data collected by the Born in Bradford cohort. The focus of analysis was migration status groupings, based on self-reported country of birth of participants and their parents and grandparents. Chi-Square, one-way ANOVA and correlation coefficients examined relationships between variables.

**Results:**

Migrant women were less likely to smoke (native: 34.4%, 1st generation: 2.8%, 2nd generation: 8.6%) or to be obese (native: 25.5%, 1st generation: 17.4%, 2nd generation: 21.3%) compared to native women. Migrants were less physically active at 6 months (Mean (SD) minutes/week: native 265 (245), 1st generation 113 (162), 2nd generation 147 (182)) with larger increases in BMI over time compared to native women. Migrant women were more likely to be suffering psychological distress at baseline and 6 months postpartum and migrant families were more likely to live in areas of high socio-economic deprivation, despite higher levels of educational attainment.

**Conclusions for Practice:**

This study ethnicity and migration identifies some important differences between ethnic groups with different migration histories, therefore indicating that healthcare professionals should consider eliciting full migration histories to improve care. The impact of these differences on perinatal outcomes is a priority for future research.

## Significance

*What is Already Known on this Subject?* There is substantial ethnic variation in health inequalities and health-related behaviours during pregnancy. Women of non-white ethnic origin are less likely to engage in harmful behaviours such as smoking or binge drinking in the UK, yet often have poorer pregnancy outcomes.

*What does this Study Add?* This study adds the dimension of migration status to the investigation of ethnicity and perinatal health. The study considers differences between first-and-second-generation migrant women and native women with no family migration history. The results show that, among women of South Asian ethnicity, women’s own country of birth and that of their parents makes a difference to patterns of health-related behaviours. This new information is important in directing health promotion activities effectively.

## Introduction

There is evidence to suggest that migrants of South Asian origin living in Europe are at increased risk of acquiring non-communicable diseases such as diabetes compared to populations in their country of origin. In pregnant populations, Jenum et al. (2012) reported a higher incidence of gestational diabetes in ethnic minority women in Norway after adjustments for age, parity and pre-pregnancy body mass (BMI). However, further adjustment for height, education, family history of diabetes and ethnicity, had little impact on gestational diabetes. A recently published study by (Li et al., 2018) on the impact of infant ethnicity and mother’s country of birth on infant mortality found a significantly higher crude mortality rate in infants whose mothers were born outside of the UK; however, this association was no longer significant after adjustment for maternal age, deprivation and marital status. After additional adjustment for gestational age at delivery, South Asian ethnic groups had significantly higher infant mortality rates compared to other groups. This association was most pronounced among Pakistani women where the odds of infant death were more than twice that of white British infants (aOR 2.32, CI 2.15–2.50). While the impact of ethnicity on health has been documented the majority of studies of this type are conducted in Western countries and do not examine the role of place of birth or length of stay in host countries for first-generation migrants. The relationship therefore between migration status, health related behaviours and issues such as access to health care is less clearly understood (Puthussery, 2016). There is some evidence to suggest that these differences may be partly attributable to acculturation and the adoption of unhealthy lifestyle behaviours more common in Western Cultures (Davies, Blake, and Dhavan, 2011). The concept of acculturation in psychology is a process of cultural and psychological change that arises from sustained contact between people of different cultural backgrounds (Berry, 2006). The inclusion of acculturation as a variable in social and health research is contentious, as the nuances of the process may be missed, leading to inconsistent or incorrect results (Hunt, Schneider, and Comer, 2004). Researchers from the United States have thus far conducted the majority of research into acculturation, with a focus on South and Central American migrants, which therefore may not be applicable in the UK context.

In the maternity population, a study of lifestyle related risk factors in the Born in Bradford cohort has established significant differences between ethnic groups (Cooper et al., 2013). This study showed that South Asian women were less likely to smoke or binge drink during pregnancy, however levels of obesity were similar across groups. While these are important findings the study did not take into account any potential intergenerational differences between UK-born and foreign-born women. A further study looking at the detection of common mental health problems in the Born in Bradford cohort found that while General Health Questionnaire scores indicating psychological distress in the baseline Born in Bradford questionnaire were higher among Pakistani women, twice as many white British women had an identified common mental health disorder (Prady et al., 2016). This may suggest a level of missed diagnoses among ethnic minority women or having different coping strategies. This study also focused on ethnicity as opposed to migration history, which is an important consideration regarding disparities in mental health trends.

The Born in Bradford cohort has collected detailed data relating to participant’s personal and family history of migration. This presents a unique opportunity to explore how health related behaviours might differ not only by ethnicity but also by migration status. This study therefore aims to investigate differences in the lifestyle and health behaviours among British born women both with and without family history of migration and women who have themselves migrated to the UK from abroad.

## Methods

Born in Bradford (BiB) is a prospective cohort study recruiting participants during pregnancy in the Northern English city of Bradford. Women were invited to participate in the Born in Bradford study when attending an oral glucose tolerance test at 26–28 weeks’ gestation or when attending other antenatal appointments. Informed consent was obtained, and women were asked to complete a baseline questionnaire providing data on maternal characteristics. Women also provided venous blood and urine samples and umbilical cord blood samples at birth. Recruitment took place between March 2007 and December 2010, and over 80% of women eligible in this period agreed to take part, which represents approximately 64% of the births occurring in Bradford during this period (Wright et al., 2012). BiB1000 is a sub-sample of this cohort, originally designed to investigate the determinants of childhood obesity, in which women and their children provided data until the infant was three years of age (Bryant et al., 2013). Data for the BiB1000 study were collected post-partum at 6 months, 12 months, 18 months, 2 years and 3 years. This study utilises data from the baseline BIB questionnaire collected during pregnancy and the 6-month and 12-month postpartum questionnaires collected as part of the BIB 1000 study to examine differences in health-related behaviours and characteristics by maternal migration status.

The variables selected for inclusion in the current study differ at different time periods due to differences in the questionnaire instruments used affecting the availability of data. Full versions of the BIB baseline questionnaire and the 6 and 12-month post-partum BIB 1000 questionnaires are available from https://borninbradford.nhs.uk/research/documents-data/.

### Migration Groups

Migration status groups were calculated using questionnaire responses to questions about the women’s own country of birth and that of her parents and grandparents. First-generation migrants are therefore those women who were themselves born outside of the UK; second-generation migrants those women who were themselves born in the UK but have at least one parent who was born abroad. The second-generation migrant group also includes those with a higher order migrant background (i.e. grandparents born abroad) due to small numbers, precluding further stratified analysis. Women in the native group were those who reported that they themselves, their parents and their grandparents were all born in the UK, regardless of self-reported ethnicity. While women’s ethnicity did not have a bearing on group allocation, the analysis explores the impact of ethnicity as an independent variable.

### Outcome Variables

The current study focuses on exploring differences between groups based on migration status in individual variables that impact on maternal and infant health and wellbeing. The study utilises the most relevant variables from each of the three questionnaire data sets, with changes over time examined where adequate comparable data were available. For first-generation migrants this includes length of stay in the UK at baseline. The variables included in the study from the baseline questionnaire were Body mass index (BMI); smoking history; smoking during pregnancy; second-hand smoke exposure; use of vitamins and supplements and General Health Questionnaire-28 (GHQ-28) score. Variables included from the BIB 1000 six-month postpartum questionnaire were BMI; current smoking; smoke exposure; fruit and vegetable consumption; GHQ-28 score; breastfeeding initiation and physical activity. Variables included from the BIB 1000 twelve-month postpartum questionnaire were BMI; and Kessler Scale-6 (KS6) score.

BMI was calculated using weight divided by height squared (kg/m^2^). For comparison of BMI categories, international cut off values published by the World Health Organisation were used (WHO Expert Consultation, 2004). The GHQ-28 is a validated instrument for identifying minor psychiatric disorders in the general population. Answers are given on a four-item scale and scored using the recommended GHQ scoring system 0-0-1-1 and a higher score indicates a higher level of psychological distress. The analyses of GHQ-28 uses data medians and non-parametric tests, due to a significant skew in the data. GHQ-28 score above the 75th centile for the sample indicates psychiatric disorder (Prady et al., 2013).

### Statistical Analysis

The focus of this study is to detect differences between groups of individuals with different migration status. The study population was therefore categorised into three groups for analysis according to maternal country of birth and that of their parents and grandparents: native, first-generation migrant or second-generation migrant. Statistical analysis was undertaken using SPSS 24.

### Analysis of Differences Between Migrant Groups

The bivariate analysis of demographic and health related variables used Chi-Square (for categorical variables) and one-way ANOVA (for continuous data); categorical variables are presented as percentages and continuous variables as means and standard deviations.

### Length of Stay

For first-generation migrants, the analysis calculated length of stay in the UK from the woman’s self-reported age of arrival in the UK and her age at the time of delivery. Analysis of the impact of length of stay in this group used correlations between length of stay and BMI and GHQ score and reported as a correlation coefficient (R) and its associate P value.

## Results

### Demographics at Baseline

The demographic characteristics of the sample vary considerably between groups. Native women have the highest proportion of pregnancies among teenagers while the rate of pregnancies in those aged over 35 is highest among first-generation migrants. First-generation migrants had substantially higher parity, with 19% having parity of three or more compared to 15.5% of second-generation migrants and 7.2% of natives. Both first- and second-generation migrants were much more likely to be married and less likely to be single parent compared to native women.

Both the mothers and fathers of children born to migrant women had a higher level of educational attainment compared to natives. Only 9% of native women had never had a paid job at baseline compared to over half of first-generation migrant women who had never done paid work outside of the home. The differences in employment patterns among fathers were less striking. There was a higher rate of unemployment (including students) among native men compared to both migrant groups and higher levels of self-employment among migrants. Despite higher levels of education, migration status appears to be linked to higher levels of deprivation according to index of multiple deprivation (IMD). IMD is the official measure of relative deprivation for small areas in England and combines information from seven domains of deprivation (income, employment, education, health, crime, housing and environment) to give a deprivation score (Office for National Statistics, 2020). Table 1 shows a summary of demographic variables across the sample.

**Table 1:** Characteristics of the sample at baseline

	Native		First generation migrant		Second or higher order generation migrant		Unknown			Total		
	N	%	N	%	N	%	N	%		N	%	p=
Whole Cohort	4397	38.40	4201	36.70	2840	24.80	7	0.10		11,445	100	
Ethnic Group												
Asian or Asian British	11	0.3	3435	81.8	2499	88.0	0	0		5945	51.9	< 0.001
White or White British	4273	97.2	350	8.3	184	6.5	7	100		4814	42.1	
Black or Black British	8	0.2	199	4.7	38	1.3	0	0		245	2.1	
Mixed ethnic group	100	2.3	22	0.5	104	3.7	0	0		226	2.0	
Chinese	0	0	77	1.8	4	0.1	0	0		81	0.7	
Other	3	0.1	112	2.7	9	0.3	0	0		124	1.1	
Language used to administer questionnaire												
English	4383	99.7	2189	52.1	2791	98.3	7	100		9370	81.9	< 0.001
Mirpuri or Punjabi	1	0.0	544	12.9	9	0.3	0	0		554	4.8	
Urdu	1	0.0	1444	34.4	31	1.1	0	0		1476	12.9	
Other	0	0.0	10	0.2	0	0.0	0	0		10	0.1	
Maternal age group												
15–19	515	11.7	82	2.0	128	4.5	0	0		725	6.3	< 0.001
20–34	3334	75.8	3555	84.6	2368	83.4	6	85.7		9263	80.9	
35+	548	12.5	564	13.4	344	12.1	1	14.3		1457	12.7	
Marital Status												
Married	1372	31.2	3771	89.8	2381	83.8	2	28.6		7526	65.8	< 0.001
Not married - living with partner	1715	39.0	176	4.2	130	4.6	2	28.6		2023	17.7	
Single	1301	29.6	246	5.9	323	11.4	3	42.9		1873	16.4	
Parity												
0	2009	48.6	1332	33.9	1073	40.4	2	28.6		4416	41.2	< 0.001
1	1293	31.3	1089	27.7	704	26.5	2	28.6		3088	28.8	
2	539	13.0	764	19.4	466	17.5	3	42.9		1772	16.5	
3+	296	7.2	745	19.0	413	15.5	0	0		1454	13.6	
Mother’s highest level of education												
Less than 5 GCSEs grade A-C or equivalent	892	20.3	1157	27.5	417	14.7	2	28.6		2468	21.6	< 0.001
5 GCSEs grade A-C or equivalent	1533	34.9	1078	25.7	892	31.4	0	0		3503	30.6	
A-levels or higher	1553	35.4	1687	40.3	1328	46.8	3	42.9		4571	40	
Other/unknown	415	9.4	260	6.2	199	7.0	2	28.6		876	7.7	
Father’s highest level of education												
Less than 5 GCSEs grade A-C or equivalent	768	17.5	603	14.4	379	13.4	1	14.3		1751	15.3	< 0.001
5 GCSEs grade A-C or equivalent	1155	26.3	850	20.3	732	25.9	2	28.6		2739	24	
A-levels or higher	1158	26.4	1765	42.2	1117	39.5	2	28.6		4042	35.4	
Other/unknown	1312	29.9	963	23.0	601	21.2	2	28.6		2878	25.2	
Mother’s employment status												
Currently employed	2766	62.9	986	23.5	1284	45.2	5	71.4		5041	44	< 0.001
Never employed	396	9.0	2258	53.7	466	16.4	0	0		3120	27.3	
Previously employed	1232	28.0	944	22.5	1087	38.3	2	28.6		3265	28.5	
Father’s employment status												
Employee	3199	77.3	2947	72.6	1963	71.5	5	83.3		8114	74.1	< 0.001
Self-employed	422	10.2	678	16.7	542	19.8	0	0		1642	15	
Unemployed/student	516	12.5	433	10.7	239	8.7	1	16.7		1189	10.9	
BMI (kg/m^2^)												
Underweight	101	2.5	209	5.5	121	4.6	0	0		431	4.1	< 0.001
Normal weight	1744	43.7	1808	47.3	1188	45.4	1	16.7		4741	45.4	
Overweight	1131	28.3	1139	29.8	747	28.6	3	50		3020	28.9	
Obese	1019	25.5	663	17.4	558	21.3	2	33.3		2242	21.5	
Ever smoked (Yes)	2595	59	310	7.4	578	20.4	4	57.1		3487	30.5	< 0.001
Smoking during pregnancy (Yes)	1513	34.4	117	2.8	243	8.6	2	28.6		1875	16.4	< 0.001
Smoke exposure (Yes)	950	21.6	286	6.8	289	10.2	1	14.3		1526	13.3	< 0.001
Any supplements in last 4 weeks (Yes)	1334	30.3	2026	48.2	1353	47.6	2	28.6		4715	41.2	< 0.001
	**N**	**Mean (SD)**	**N**	**Mean (SD)**	**N**	**Mean (SD)**	**N**	**Mean (SD)**		**N**	**Mean (SD)**	**p=**
IMD Score	4395	36.60 (19.2)	4200	47.04 (15.5)	2840	43.82 (16.3)	7	46.75 (21.2)		11,442	42.23 (17.8)	< 0.001
Number of weeks gestation at booking appointment	4007	12.18 (2.83)	3781	12.88 (3.17)	2570	12.43 (3.12)	7	11.41 (2.63)		10365.0	12.50 (3.01)	< 0.001
BMI	4056	26.8 (6.0)	3885	25.3 (5.2)	2650	26.0 (5.7)	7	28.5 (5.2)		10,598	26.0 (5.7)	< 0.001

### Outcome Variables

Results relating to the outcome variables are presented at three time points: baseline during pregnancy, six months postpartum and 12 months post-partum.

**Fig. 1:**
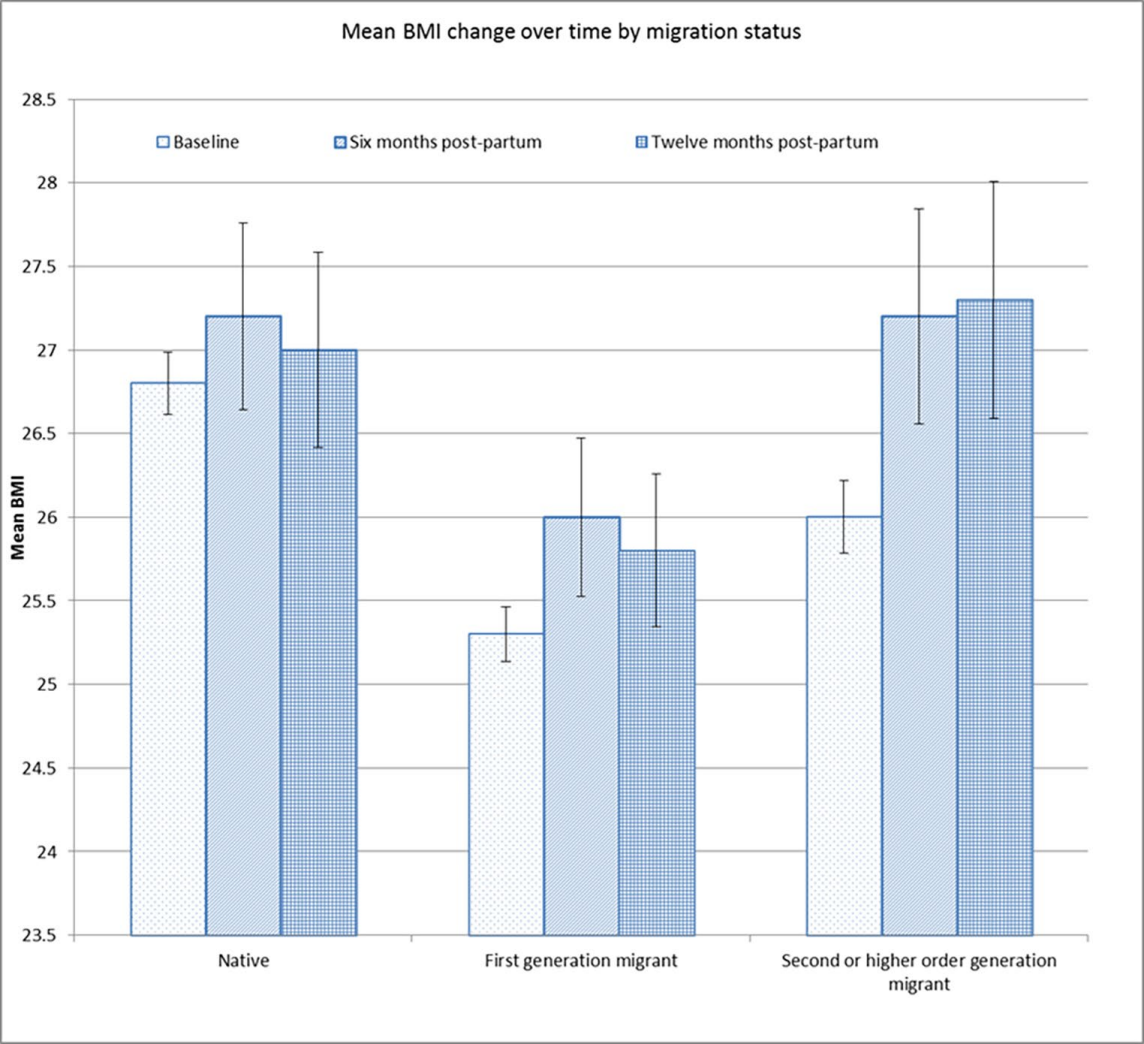
Mean BMI change over time by migration status

At all time points, there was a higher proportion of underweight and a lower rate of obesity among first-generation migrants compared to the other two groups. Maternal BMI at booking was also positively correlated with length of stay for first-generation migrants (R = 0.218, P = < 0.001). Mean BMI increase was higher among migrant women from baseline to 6 and 12 months postpartum compared to the native women (Fig. 1). Native women were substantially more likely to smoke at all time points, including during pregnancy (Table 1).

**Fig. 2:**
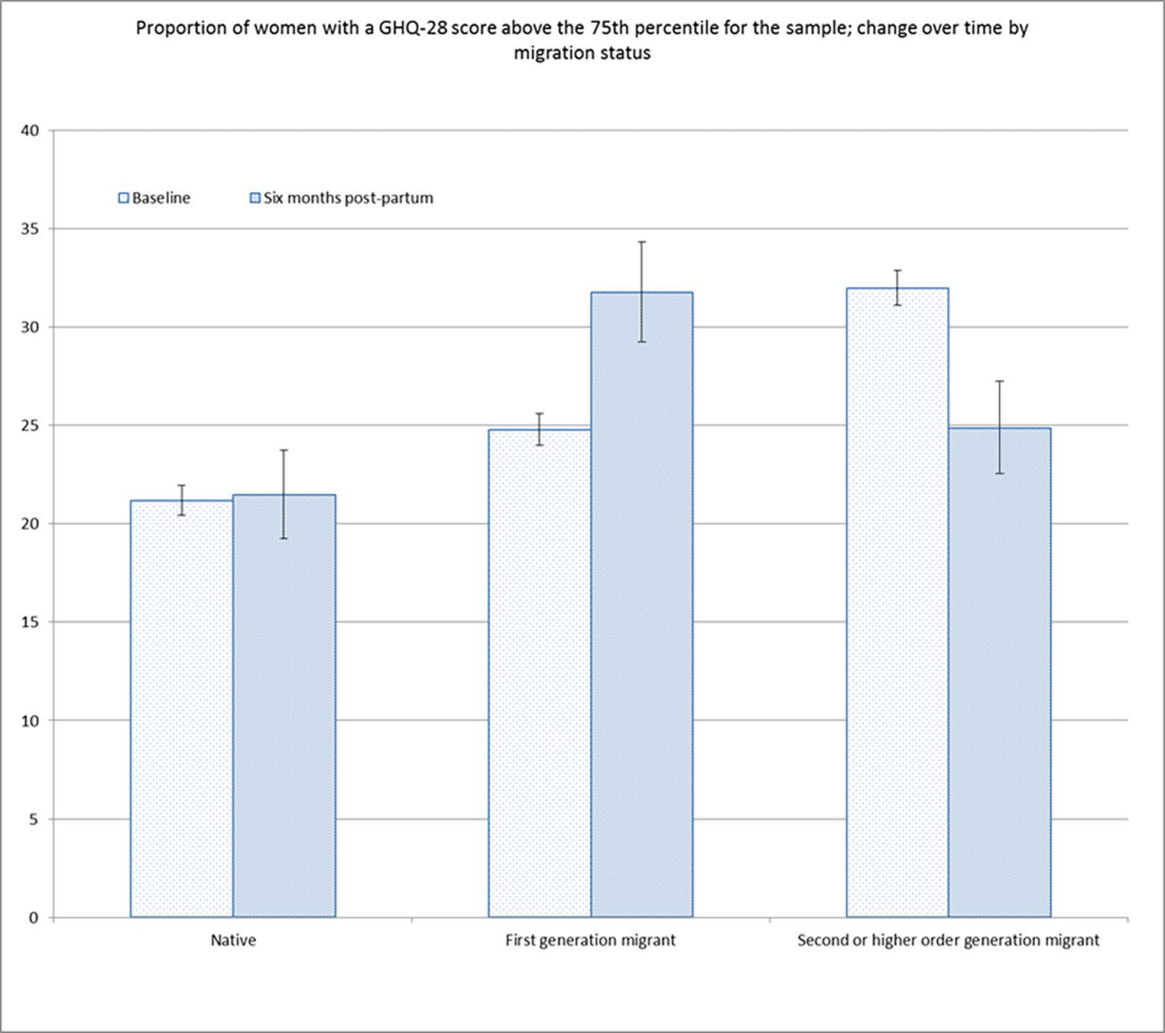
Proportion of women with a GHQ-28 score above the 75th percentile for the sample; change over time by migration status

Analysis of the General Health Questionnaire as an indicator of mental health was based on the median value with thresholds indicating likely psychological distress set at above the 75th percentile for the sample. This showed that at baseline the median in second-generation migrants was significantly higher than that in the other two groups; however, at 6 months postpartum both migrant groups had a higher median score compared to non-migrants. For first-generation migrants, length of stay in the UK was positively correlated with increasing GHQ score at baseline (R = 0.101, P = < 0.001) and score difference from baseline to 6 months postpartum (R = 0.161, P = < 0.001). This relationship was not apparent however in the total score at six months postpartum which showed a slight negative correlation, although this wasn’t statistically significant (R=-0.059, P = 0.199). Figure 2, shows the results of the analyses of GHQ score baseline and six months postpartum by migration status. At twelve months postpartum psychological distress was measured using the shorter Kessler Scale (6) meaning that these results are not directly comparable with those at earlier time points; there were no significant differences in Kessler Scale score by migration status.

**Fig. 3:**
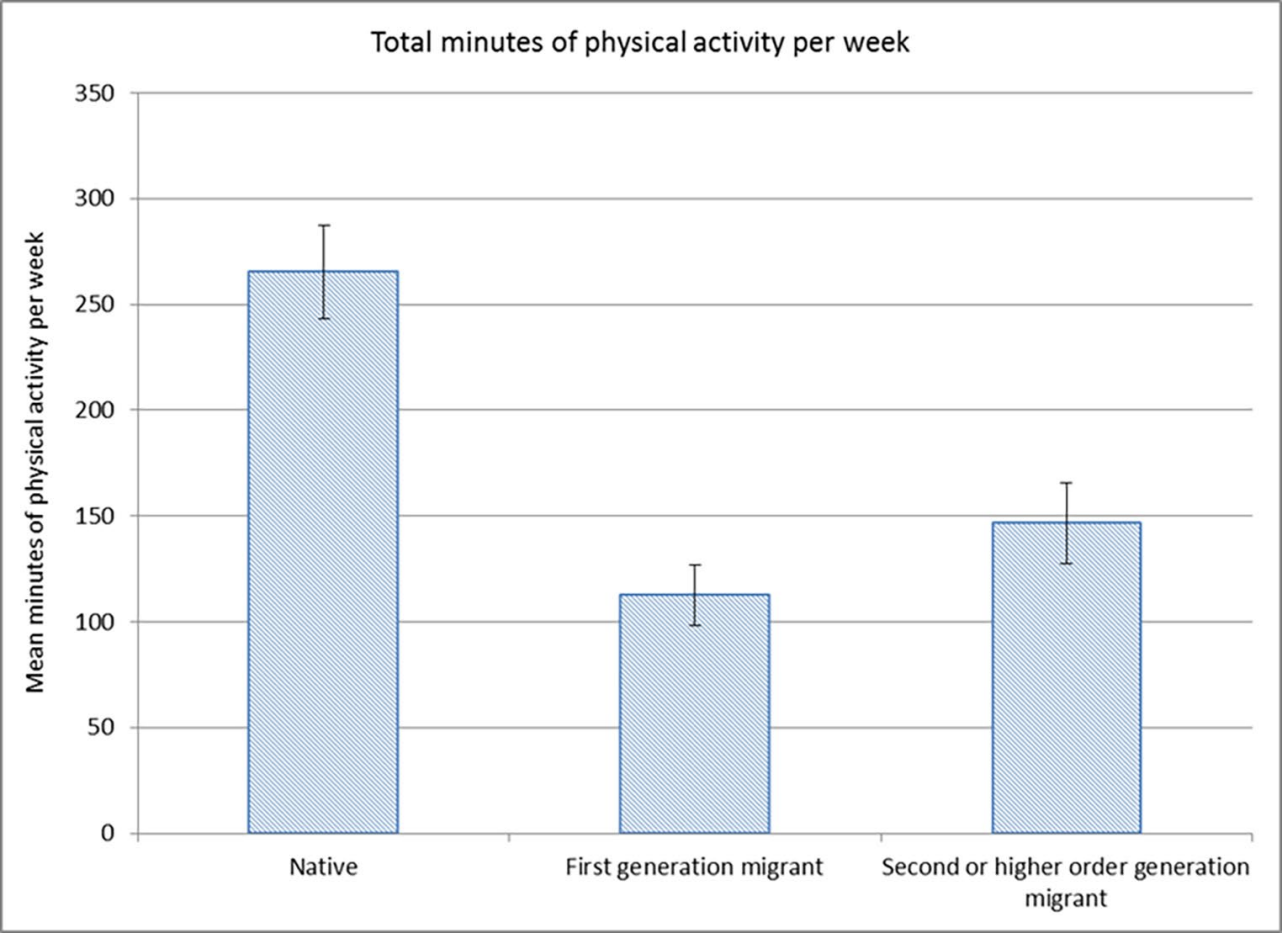
Mean number of physically active minutes per week at six months postpartum by migration status

Maternal physical activity data were only collected at 6 months postpartum. There were significant differences between groups in mean total minutes of physical activity per week with native women reporting more than double the number of active minutes compared to first-generation migrants, as shown in Fig. 3.

## Discussion

The current study aimed to investigate the basic characteristics, lifestyle and health related behaviours of a large, diverse cohort of women during pregnancy and up to a year postpartum with a focus on maternal migration status. The self-reported country of birth of the mother and of her parents and grandparents informed migration status groups. While ethnicity was not the focus of this study the majority of women in both the first-generation migrant and second-generation migrant groups were of South Asian ethnicity with the majority of these women’s heritage being in Pakistan.

The exploration of the demographic characteristics of the sample showed substantial differences in age and parity for first-generation migrants compared to native women, suggesting different care needs. Higher levels of maternal education and yet lower rates of employment and higher IMD as an indicator of area deprivation among migrant women is also of interest and warrants further in-depth exploration.

The native women were more likely to become mothers during adolescence whereas a higher proportion of migrant women were continuing to have children at older ages. Higher rates of teenage pregnancy in the native communities as opposed to migrant populations is of interest. A higher rate of women being a single parent or living without a partner in native women is also of concern, meriting in depth explorations.

Other positive health related behaviours in migrant women included lower rates of smoking and higher rates of breastfeeding. This is particularly of interest as the results suggest a trend towards rates seen in the native population in second- generation migrants. These findings potentially add evidence to the theory of acculturation and adapting the host country behaviours albeit with potential health and social complications. These findings relate to the concept of the ‘Healthy Migrant Effect’, where migrant populations have better health outcomes relative to native populations. This concept has come under scrutiny, particularly in the UK, as self-reported ethnicity is often assumed as a proxy due to a lack of data relating to migration status and country of origin in health datasets. Mortality rates in the 2011 census show a slight advantage in terms of life expectancy at birth among first-generation migrants. The authors concluded that improving recording of ethnicity, country of origin and migration status in health records is of paramount importance in effectively studying the patterns of health and disease in migrant and ethnic minority populations. The ability in this study to assess migration status in addition to ethnicity is therefore important; however further work is needed to understand how these observations might translate into differences in perinatal outcomes.

While overweight and obesity are higher in non-migrant populations, migrant women had higher BMI increases perinatally may suggest some impact of acculturation on eating habits. The correlation between length of stay in the UK and BMI for first-generation migrants in our study appears to support this. While there has been some debate regarding amending BMI classifications based on ethnicity, particularly for Asian populations, this study uses the international classifications outlined by the World Health Organisation. This approach was recommended by a WHO expert consultation published in the Lancet (WHO Expert Consultation, 2004), along with a recommendation to consider additional action points in the continuum of BMI for South Asians reflecting a higher incidence of metabolic disturbances at lower BMIs in this population.

The results also suggested a higher likelihood of psychological distress and mental health problems in migrant groups. Our study also shows that this may worsen as length of stay in the UK increases, assessed by the positive correlation of length of stay with increasing GHQ-28 score and a higher proportion of women scoring over the 75th percentile for the sample in the second-generation migrant group. A review of ethnic minority women’s experiences of perinatal mental health conditions and services in Europe found that women reported significant barriers to recognising and seeking help for mental health problems (Watson et al., 2019). Women reported cultural expectations and ongoing stigma as barriers to help seeking and those who did seek help reported difficulties with fractured and culturally incompetent services. Understanding the burden of perinatal mental ill health in migrant and ethnic minority populations and developing appropriate interventions is a significant research priority. Previously established differences in maternity care access and utilisation among migrants, particularly those who have arrived recently in the UK, compounds the urgency of this area of research. A systematic review of migrant women’s experiences of maternity care highlighted the need for culturally competent and trauma-informed care underpinned by good continuity of care and interdisciplinary work to support the unique needs of migrant women, particularly those who are newly arrived (Fair et al., 2020).

There have been criticisms of the concept of acculturation, particularly in its application to health research. (Fox et al., 2017) argue that inconsistencies in health disparities observed in relation to indicators of acculturation exist due to the complexities of acculturation as a concept and the fact that the biological effects of factors such as values, behaviours and cultural change will vary wildly. It is therefore key to understanding differences in population groups to consider a wide range of potential contributory factors as opposed to attempting to create one summery variable to represent levels of acculturation. Methodological research relating to models of acculturation applicable to public health research are lacking; where this does exist, Latino migrants in the USA are the focus (Abraído-Lanza et al., 2006). This study is one of the few studies attempting to unravel the impact of migration and ethnicity in pregnant women and new mothers in order to add much needed insight to this debate. Identifying where disparities in factors associated with health outcomes lie enables further analysis of adverse outcomes, and informs development of appropriate interventions for enhancing perinatal health outcomes.

## Conclusion

This study presents valuable new information regarding the determinants of perinatal and child health for different migrant groups. There is evidence to suggest that basic characteristics and health related behaviours are more positive in first-generation migrants and that there is some acculturation affect bringing second-generation migrant women closer to native women in terms of health behaviours. This study also presents evidence of higher levels of psychological distress in migrant women which is not reflected in formal diagnoses of mental health conditions. This highlights an area of work which warrants urgent attention. Further work to explore the interplay between health behaviours, maternal ethnicity and migration status and their effect on pregnancy and birth outcomes is critical to establish where targeted interventions might benefit communities.

## Data Availability

requests for data access should be made directly to the data owner, Bradford Royal Infirmary NHS Trust. Details regarding data access are available from https://borninbradford.nhs.uk/research/how-to-access-data/.
